# Six weeks that changed the preterm infant brain: lessons learned from the Family Nurture Intervention randomized controlled trials

**DOI:** 10.3389/fpsyg.2024.1374756

**Published:** 2025-01-07

**Authors:** Robert J. Ludwig, Michael M. Myers, Martha G. Welch

**Affiliations:** ^1^Department of Pediatrics, Columbia University Medical Center, New York, NY, United States; ^2^Martha G Welch Center, New York, NY, United States

**Keywords:** attachment, NICU intervention, brainstem, autonomic theory of emotions, emotional connection, instinct, mother-infant, skin-to-skin care

## Abstract

**Aim:**

We review extensive results from two randomized controlled trials conducted over 9 years, comparing standard care (SC) in level-4 neonatal intensive care units (NICUs) with SC plus Family Nurture Intervention (FNI).

**Methods:**

FNI included ~six weeks of facilitated mother-infant interactions aimed at achieving mother-infant ‘autonomic emotional connection’, a novel construct that describes the emotional mother-baby relationship at the level of the autonomic nervous system.

**Results and conclusion:**

Thus far, 18 peer-reviewed publications documented significant positive short-and long-term effects of FNI on infant neurobehavioral functioning, developmental trajectories and both mother and child autonomic health through five years. The observed profound effects of FNI on central and autonomic nervous system function following a relatively short intervention support a novel *autonomic theory of emotions*. We discuss the theoretical and clinical advances that grew out of the trials and speculate on how FNI changes the mother-infant relationship from ‘dysregulation’ to autonomic emotional co-regulation. We review new constructs and tools that can be used to view and measure the mother-infant autonomic emotional relationship. We present a simple blueprint to improve preterm birth outcomes. Finally, we discuss the significance of our findings and possible impact on the future of preterm infant care worldwide.

## Introduction

Together with her team, author Welch designed the Family Nurture Intervention for the Neonatal Intensive Care Unit (FNI-NICU) based on a mother–child intervention she developed during her 30-year private clinical practice from 1970 to 2000, which is described in her 1988 book, Holding Time (Welch, 1988). The therapeutic intervention proved suitable for mothers and children with a wide range of behavioral problems (Welch, 1987; Welch and Chaput, 1988; Welch et al., 2006). The description and terminology of FNI-NICU differs from Welch’s past work. However, the key mother-infant emotional exchange activity encouraged during FNI-NICU and the intervention objectives are the same.

Between 2008 and 2012, our group tested the efficacy of FNI-NICU in a single site randomized controlled trial (RCT) in a level-4 NICU. Between 2016 and 2019 we conducted a multisite replication RCT of FNI-NICU in two level-4 NICUs serving disparate populations in two different states. FNI promoted biobehavioral activities that enhanced the mother’s and infant’s sensory experiences of each other via mutual scent exchange, face-to-face contact, vocal communication, and skin-to-skin care. We expanded preterm infant neurodevelopment testing beyond that of previous research to include outcomes related specifically to the mother-infant autonomic emotional relationship and mother-infant autonomic physiology (Welch et al., 2012) and novel analyses of infant brain activity and development (Yrjola et al., 2022). Our primary objective across both studies was to determine whether repeated mother-infant emotional engagement improved the infant’s developmental trajectory with respect to multiple outcomes—physiological, neurological and behavioral.

**Figure 1:**
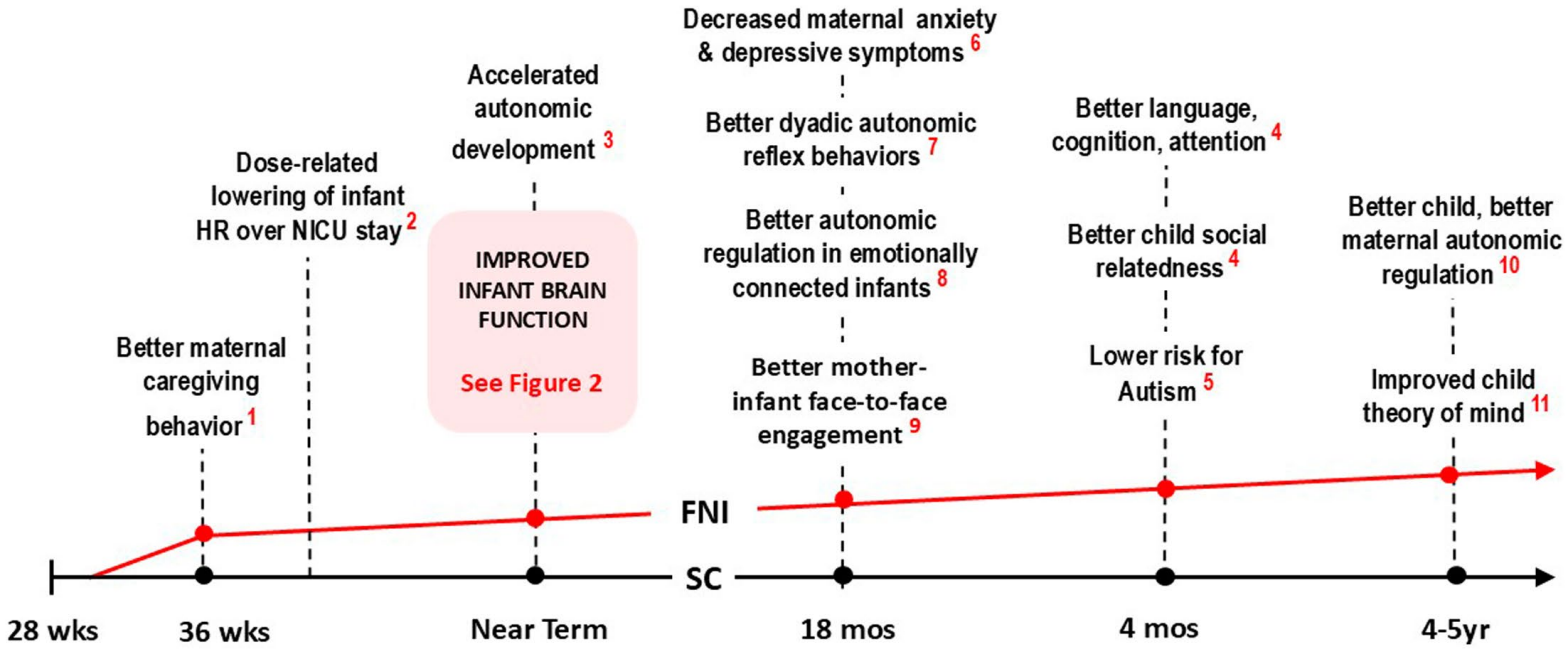
Timeline trajectory of FNI-NIU trial showing major effects of intervention at crucial stages of development. Note that at each stage, behavior and physiology were, on average, significantly better in the FNI group (indicated graphically by the red line), compared to the SC group (black line). Collectively, the results indicate that that infants in the FNI group were on a significantly better developmental trajectory following a relatively short intervention in the NICU (References: 1. Hane et al., 2015; 2. Ludwig et al., 2021; 3. Porges et al., 2019; 4. Welch et al., 2016; 5. Hane et al., 2019; 6. Beebe et al., 2018; 7. Welch et al., 2015; 8. Welch et al., 2020a; 9. Firestein et al., 2022).

To date, we have published over 18 papers comparing groups receiving Standard Care (SC) with groups receiving Standard Care plus FNI (see Figure 1). Compared to controls, analyses showed that FNI infants had significantly improved neurobehavioral and autonomic function, FNI mothers showed significant improvement in depressive symptoms and the FNI-NICU dyads scored higher on relational health measures at key assessment points through age five.

Key short-term findings between the start of the intervention in the NICU stay to immediately following discharge included improved maternal caregiving behavior (Hane et al., 2015), a lower HR over the course of the NICU stay (Ludwig et al., 2021) and better infant autonomic regulation at term age (Porges et al., 2019).

**Figure 2:**
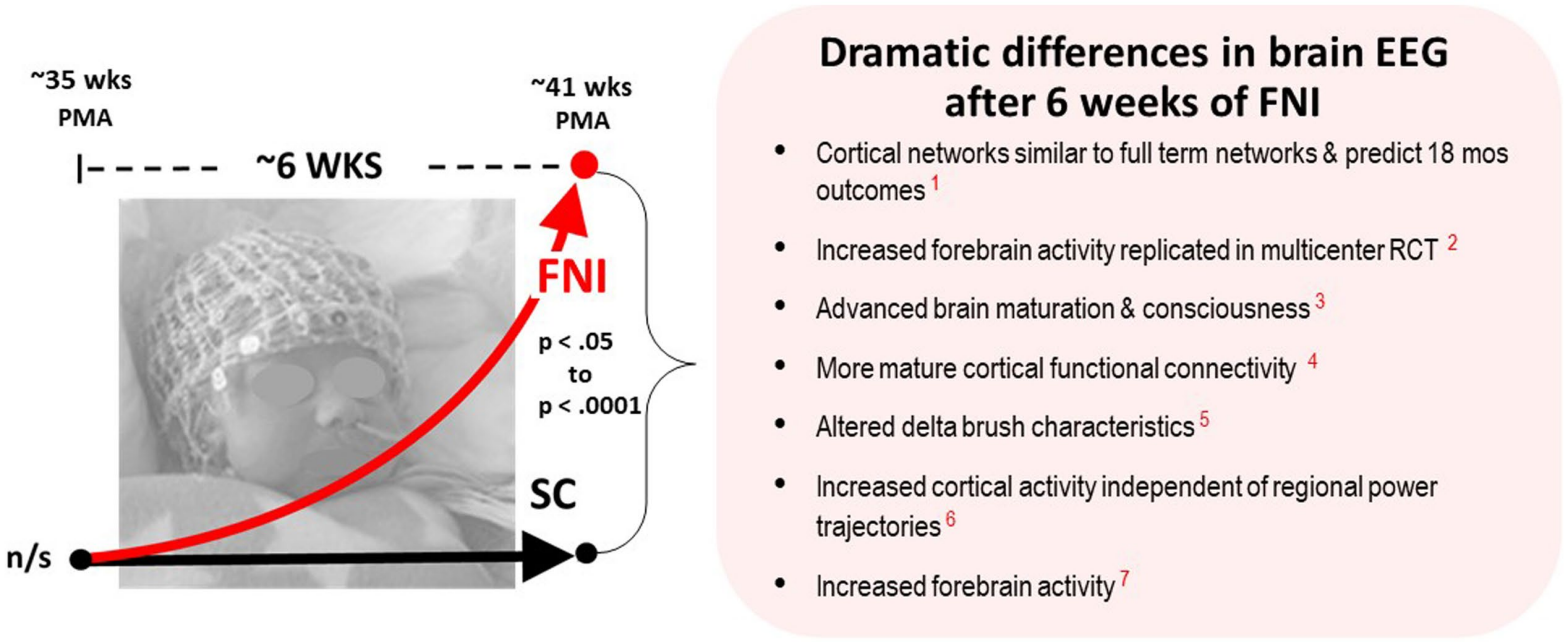
Schematic showing the dramatic changes in brain EEG following an average of six weeks intervention. Recorded at ~35 wks and ~ 41 postmenstrual age (PMA) using high density 128-lead nets. The richness of the data set and volume of EEG technical analyses, along with the p values and effect sizes, provide highly significant evidence that FNI had a very large beneficial impact on infant brain development—prompting the questions: *What exactly is the intervention?*, and *What mechanisms could explain these changes?* (Published EEG results referenced are in red: 1. Yrjola et al., 2022; 2. Welch et al., 2022; 3. Isler et al., 2018; 4. Myers et al., 2015; 5. Welch et al., 2020b; 6. Welch et al., 2017; 7. Welch et al., 2014) (Published results referenced are in red).

The centerpiece of FNI infant brain function studies were comparisons of electroencephalographic (EEG) recordings collected at ~35 weeks gestational age and ~ six weeks later at ~term age (Figure 2). Seven publications documented dramatic changes in the brain function of FNI infants, including: Increased forebrain activity (Welch et al., 2014), Increased cortical EEG activity independent of regional power trajectories (Welch et al., 2017), altered EEG delta brush characteristics (Welch et al., 2020b), more mature cortical functional connectivity (Myers et al., 2015), advanced brain maturation & consciousness (Isler et al., 2018), increased forebrain EEG activity which was replicated in our multicenter RCT (Welch et al., 2022), and cortical EEG networks that were similar to full term networks and which predicted better 18-month outcomes (Yrjola et al., 2022).

Longitudinal findings included better infant autonomic regulation following a mother-infant social stress paradigm (Hane et al., 2019), better dyadic autonomic reflex behaviors at 4-month follow-up (Hane et al., 2019), better mother-infant face-to-face engagement (Beebe et al., 2018), and decreased maternal depressive symptoms and anxiety at 4-month follow-up (Welch et al., 2016), better child social relatedness, language, cognition, attention and a lower risk for autism at 18-month follow-up (Welch et al., 2015), and better empathic understanding (Firestein et al., 2022), and better autonomic regulation at 4–5 year follow-up (see Figure 1; Welch et al., 2020a). FNI-NICU documented the effects of the intervention on mother and child across multiple domains over a five-year period (See Figure 2).

Additional analyses are ongoing, with several additional manuscripts still in preparation. However, at this point in the follow-up of FNI trial cohorts, we can say with confidence that the infants and mothers in the intervention group experienced significant short and long term benefits from the intervention. Additionally, the longitudinal effects through age five suggest the intervention significantly reduced neurobehavioral risks and improved developmental *trajectories*.

The significant findings from the FNI trial, together with the novelty and relatively low dose of the intervention, led us to examine the key factors that might have accounted for the results. Important questions we considered were:

- What key elements of the intervention led to the changes?
- What behavioral and physiological biomarkers were associated with the change?
- What biological mechanisms might have accounted for the change?
- Is the intervention scalable?

## Key elements of family nurture intervention

Mothers assigned to the FNI group agreed to attend at least four 1-h intervention ‘calming sessions’ per week while in the NICU. The average amount of time the mothers engaged with their babies in calming sessions was about the minimum we had required—between 24 to 36 h over an average six week stay. We encouraged other family members, including the father and grandparents to engage with the baby, especially when the mother could not be with the baby. However, all family members learned that their primary role was to support the disrupted emotional connection between mother and baby.

‘Calming sessions’ between mother and infant included skin-to-skin contact, mutual scent exchange, eye contact and oral communication. Skin-to-skin holding was encouraged as soon as possible but reassuring sustained touch of the infant’s torso to mimic holding was initiated while the baby was in the isolette. These activities facilitated by FNI-NICU are used in other NICU interventions, with the goal of improving infant and maternal outcomes. However, the key singular activity that is encouraged during the 1-to 1.5-h FNI ‘calming sessions’, one that is unique to FNI is ‘*autonomic emotional expression’* between mother and baby. To our knowledge, FNI is the only intervention that focuses narrowly on autonomic emotional *expression* as the critical factor in changing the mother/baby autonomic emotional *relationship*.

**Figure 3:**
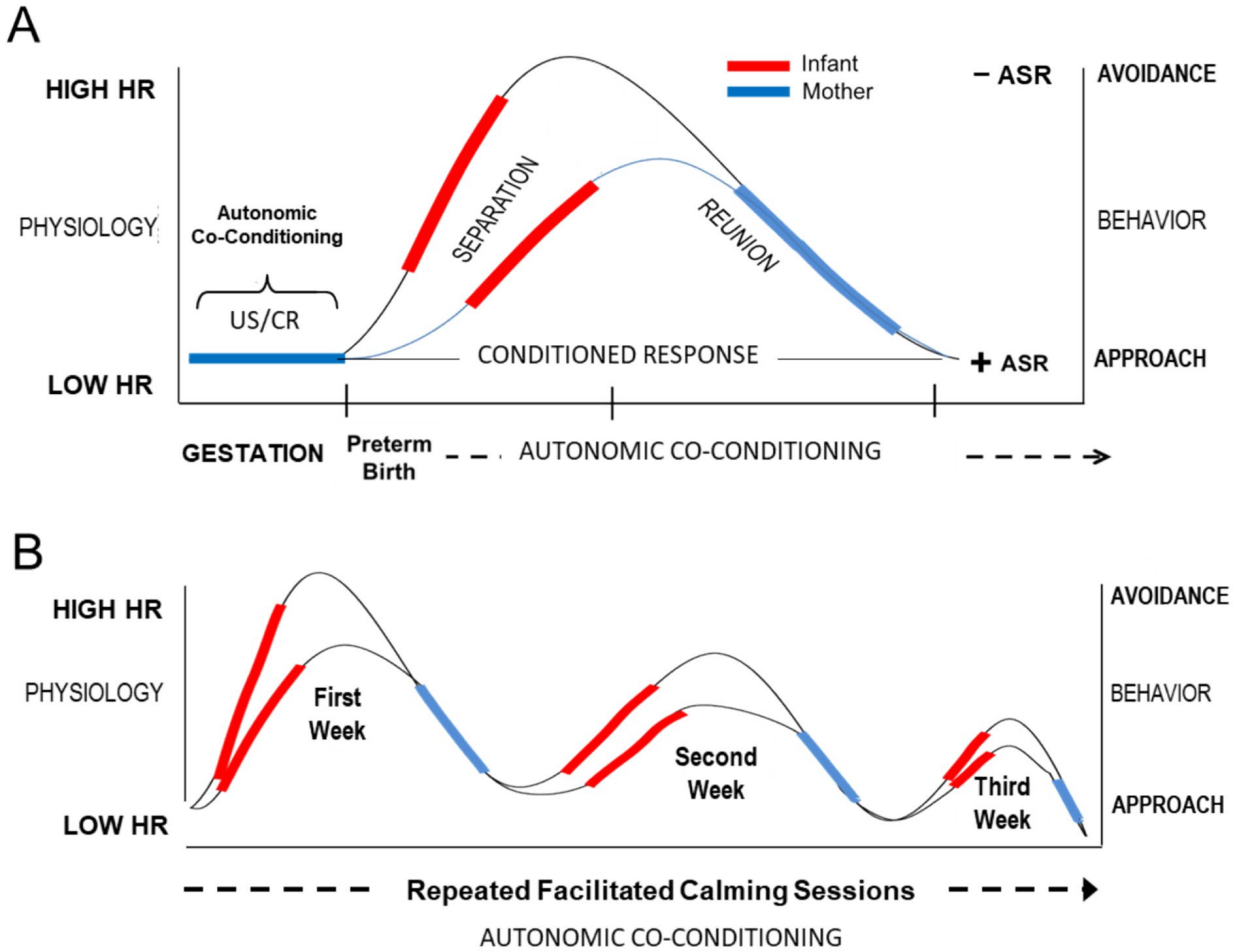
Conceptual diagram illustrating hypothesized autonomic conditioning of infant resting HR across the course of facilitated FNI calming sessions in the NICU. **(A)** Conditional autonomic reflexes (US/CRs) are formed between mother and fetus. The US/CRs are preserved transnatally in the form of *autonomic socioemotional reflexes* (ASR). Premature birth and NICU experience leads to autonomic dysregulation (e.g., increased resting HR and a negative avoidant ASR). Each facilitated calming session lowers resting HR and restores autonomic co-regulation. **(B)** Repeated facilitated calming sessions restore a positive ASR and lower resting HR, which promotes a healthy mother-infant relationship and adaptive growth and development.

**Figure 4:**
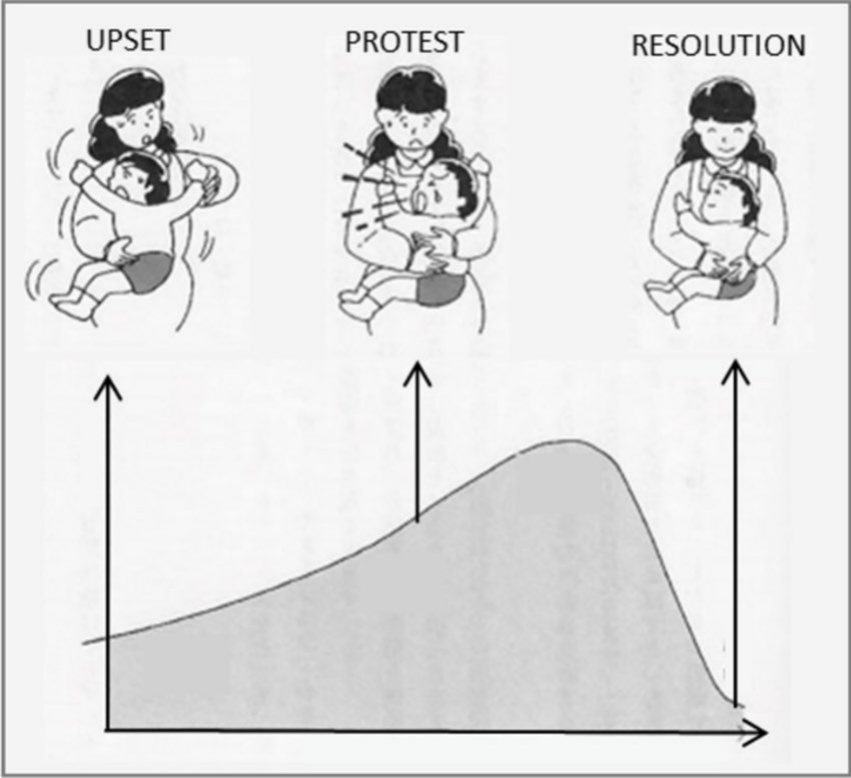
Schematic showing the hypothesized behavioral arc that results from the Welch Method.

The central mother-infant interaction of FNI was referred to as a ‘calming cycle’ (Figure 3 below). The basic phenomenon was first described in Holding Time (Welch, 1988), as schematically represented in Figure 4. The insight for the intervention came from Welch’s clinical observations early in her career. She noted in the intervention that the mother/child emotional relationship cycled through upset, protest/conflict and resolution/calm. Sometimes the cycle occurred daily, sometimes multiple times (e.g., bedtime, drop off at day care, phone call interruptions). Welch noted that the upsets were usually related to separation, or the fear of separation. In her intervention, she discovered that if the mother and child processed the upset or fear by holding and communicating with one another until the upset was resolved—and if the two repeated this process each time upset occurred—the communication between the two improved, and the upsets and symptomatic behavior occurred less often, and the pair’s emotional relationship and child behavior and development improved.

Welch designed a study to test her intervention in the neonatal intensive care unit (NICU), which came to known as Family Nurture Intervention (FNI-NICU). The main activity in the trials came to be known as a ‘calming cycle’, where the mother was encouraged to convey her heart-felt emotional communication directly to the infant. The central hypothesis for her studies was that repeated calming sessions would lead to observable positive physiological and behavioral changes in the infant and mother.

FNI-NICU focused on enabling mothers to engage in specified mother-infant emotional interactions as early as possible after birth, within the constraints of the NICU environment. Of necessity, these interactions began while the infant was confined to the isolette. Later, sessions occurred during skin-to-skin or clothed holding and near discharge during family sessions, when strategies were developed for the family members to support the mother and infant as they continued calming interactions at home. The maternal interactions principally included deeply felt *emotional expression* and firm sustained touch, vocal soothing and eye contact during the sessions, and an odor-cloth exchange at the end of each session.

### Autonomic emotional expression

What is autonomic emotional expression? It is well documented that unexpected hospitalization, and the uncertain prognosis of pre-term infants often leads mothers to experience strong negative emotions, such as frustration, anger, anxiety, even guilt (Wang et al., 2021). In response, many strategies are now in place to support and help mothers cope with the NICU experience, such as Family Centered Care (Abukari and Schmollgruber, 2023), Skin-to-skin Care (Feldman and Eidelman, 2003; Durmaz et al., 2023), developmental care (Smith et al., 2023), Interpersonal therapy (Petersson et al., 2023), and telenursing (Maleki et al., 2022). Over the past 50 years, these interventions have greatly improved the quality of care of families of preterm infants. FNI shared the goals and methods of many of these strategies.

However, the central feature of the FNI-NICU method, one that distinguishes it from all prevailing strategies, was focusing on the release and expression by the mother of her deepest and strongest emotions directly to her infant during physical contact. Doing so can be very different from how she might express emotions and other social information in contexts that make up her everyday life, especially in the NICU (Barrett et al., 2019). To avoid confusion with other types of emotional expression by the mother, the FNI-NICU trials encouraged what we now term *Autonomic Emotional Expression* (*AEE*), i.e., strong emotion expression that triggers an autonomic response, such as crying during cuddling.

Nurture specialists (NS), in this case NICU nurses, facilitated mother/infant AEE during 1-to 2-h calming sessions. Rather than attempting to teach or educate the mother about autonomic emotional expression the NS prompted the mother to engage in sensory experiences of close physical contact and AEE. For instance, in the FNI trials the first calming session typically took place at the isolette about one week after birth. During the first session, the mother was prompted to express deep emotions and feelings directly to her baby while holding the baby’s torso through the portals of the isolette. Such action typically led to eye contact and approach behaviors on the part of the baby.

Sometimes, the mother was emotionally blocked and had difficulty accessing her emotions with her baby. In these cases, the NS created privacy for the mother and baby and suggested things the mother could say directly to her baby. For instance, the NS said to the mother, tell your baby… ‘*the birth story’*, or ‘*how you felt when you became pregnant’*, or ‘*how you felt when you were told the baby was going to be premature’*, etc.

Such prompting by clinical staff and therapists is often discouraged, based on the mistaken belief that it might cause psychological harm to the mother and infant in all cases. It is conventionally believed and widely accepted that prompting a mother to display or talk about past emotional disturbances may trigger unknown negative reactions. Consequently, NICU staff typically address clinical topics or keep conversations upbeat and positive. Mothers with a history of mental illness were excluded from our studies. However, almost all of the mothers were suffering from some degree of trauma and/or depression, even those who, at first, outwardly seemed to be happy, cheerful and totally unaffected by the preterm birth experience. And nearly every mother in the study who had a heart to heart talk employing AEE with her baby reported feeling better after the talk. These were documented in a study of FNI in preschool aged children (Markowitz et al., 2023). It was rare when the AEE prompts failed to evoke a mother’s emotions, and as predicted the expression triggered a response from the baby.

FNI is trauma-informed. Approach behaviors of the infant overcome the shut-down and avoidance of the mother while the infant is hospitalized. Avoidance during hospitalization ranges from not visiting to lack of engagement even when present in the NICU or dissociative care. However, our research has shown that when mothers express deep emotions directly to the infant, the infant orients to mother, and often oxygen saturation increases (Markowitz et al., 2023). These are the markers of AEC beginning to occur. The feeling of ‘guilt’ over preterm birth in NICU mothers is often overlooked (Wang et al., 2021). One particularly effective prompt the NS used to help the mother release her emotions was to suggest to the mother that she tell her infant the story of the birth and to apologize to the baby and saying, ‘*I’m sorry for the separation and for the suffering you are going through*’. This invariably prompted an autonomic emotional response (e.g., release of tears) from the mother and the orienting response in the infant.

Crying is one of the deepest, most powerful and therapeutic emotions a mother and infant can experience together in the NICU (Craig et al., 2020; Twohig et al., 2016). It is common for the mother to hold back tears to appear stoic or “strong.” Sometimes, the mother is told not to cry by friends and staff—‘*Do not cry. Everything is going to be OK’*. Sometimes, the mother will release her emotions to the staff or family members, instead of to the baby. With FNI, crying in the presence of the infant was anticipated and welcomed. The NS told the mother to direct all her emotions to the baby and let herself cry while holding her baby, whenever and as often as she felt like it. The authentic expression of maternal emotion is a powerful initiator of connection between mother and infant and brings the dyad closer, which simultaneously stabilizes the baby’s physiology, such as heart rate, oxygen saturation, etc.

As stated, the release of emotions by the mother while holding the baby typically elicits orienting behavior, which we define as a primary *social orienting reflex* on the part of the infant (Ludwig and Welch, 2022). Orienting includes turning toward the mother, opening their eyes to look at mother, and eventual direct sustained eye contact with her. When this happens, the mother typically feels an ‘autonomic emotional’ connection to her preterm baby for the first time, with mothers reporting that they feel they and baby are sharing their feelings directly with one another. The experience of AEC is accompanied by physiological changes in mother and infant, which NSs observed resulted in mutual states of calm and comfort. The NS encouraged the mother to engage in AEC whenever touching her baby in the isolette or holding her baby during skin-to-skin or clothed holding. The NS asked the mother not to use her cell phone during the brief time she had with her baby rather she was encouraged to direct her attention and emotional feelings directly to her baby.

When interacting with the hospitalized infant, FNI mothers were instructed to speak or sing to her baby in their ‘native’ tongue (i.e., the language spoken at home when she was a child), if she knew it. This is because the emotional content of expression between the mother and baby is conveyed in the primary language, sometimes referred to as ‘motherese’ (Welch and Ludwig, 2017a; Dave et al., 2018). NICU staff and NSs reported this had several benefits for mothers. At a time when privacy is difficult to establish, the dyad was drawn into an intense interpersonal communication that mentally blocked out external noises. It also reduced the feelings of isolation for the mothers who did not speak English. Finally, our research suggests that emotions are better communicated and more easily conveyed when expressed verbally in the pair’s native language (Hane et al., 2024).

Clinical observations by the NSs and study staff confirm that once the mother released her deep emotions to the infant, the infant became emotionally available to the mother and responded behaviorally through eye contact and approach behaviors. This response from her baby prompted approach behaviors from the mother. This approach-seeking by FNI infants was evident in the NICU at 36-weeks and at 4-months with both mother and infants showing more approach-seeking behavior (Beebe et al., 2018; Hane et al., 2019).

We theorize that repeated AEE between the mother and infant over the course of the NICU stay changed the dyad’s autonomic emotional relationship which, in turn, promoted positive changes in developmental trajectories of the infant’s central and autonomic nervous system function, as well as the emotional well-being of the mother.

## Physiological assessment of FNI

### Electroencephalographic (EEG) biomarkers

During the NICU stay, the preterm infant cerebral cortex has begun to support early learning and assume critical sensory/motor functions. The prefrontal cortex is the location of our most advanced cognitive functions, including attention, motivation, and goal-directed behavior. Comparing the brain neuronal activity and development of infants receiving FNI in the NICU was the primary aim of the RCT.

A Columbia University team of EEG experts, led by author Myers, included Joseph Isler, Philip Grieve and Raymond I. “Buddy” Stark, with additional analyses performed by Sampsa Vanhatalo and his team in Finland. The EEG data was singular and groundbreaking for two reasons. At time of our first RCT, Columbia was a beta site for development of cutting-edge 128-lead (high density) EEG net technology. Therefore, the amount and quality of data collected on each infant was unprecedented. At the time, EEG was generally considered by clinicians to be a medical tool and not useful for evaluating interventions or in predicting neurodevelopmental outcomes. Indeed, Dr. Stark made a prediction at the very beginning—“*You will never show a difference in EEG after six weeks of intervention*.” After our first report, he changed his mind and became one the team’s strongest advocates.

Given the variety and quality of the EEG analyses and the metadata that accompany it, there can be little doubt the FNI EEG data will provide a rich resource for many future analyses. This was dramatically demonstrated when the Vanhatalo group performed novel network function analyses on the FNI EGG data and showed that the FNI infant EEG data was not significantly different from term-age birth network EEG data and that the FNI network function at term age predicted better 18-month neurodevelopmental outcomes (Yrjola et al., 2022). We are working to make the entire FNI EEG data set available for free download and analyses on a website.

### Heart rate and vagal tone

As summarized above, FNI-NICU aims to facilitate the emotional connection between mother and infant and establish positive conditioning of the autonomic nervous system. Accordingly, we hypothesized that we should find changes in the infant and mother autonomic regulation associated with the repeated calming cycles experience of FNI. We focused on two physiological measures that might reflect these changes. First, we assessed measures of heart rate variability (i.e., respiratory sinus arrythmia (RSA), or vagal tone) that reflect ongoing effects of parasympathetic regulation of heart rate. In addition, we also measured heart rate itself, more specifically how heart rate changes over time in the NICU with exposure to calming sessions. Three outcome papers have been published thus far.

First, we found that from about 35 weeks to term age, vagal tone increased more rapidly in FNI infants, as did the effectiveness of vagal control on heart rate, or what has been termed vagal efficiency (Porges et al., 2019). In a second paper, we extracted hourly mean heart rates from a clinical monitoring system that acquired heart rate data 24/7 over the entire NICU stay. Results from this case-matched study showed that with time and ‘dose’ of calming sessions the heart rates of FNI infants decreased more rapidly than control infants (Ludwig et al., 2021). A successful replication of this finding is currently in preparation. Together, these two papers support our hypothesis that FNI-NICU enhances infant autonomic regulation and more rapid maturation of cardiac function.

Finally, in a paper assessing vagal tone data from a long-term follow-up study out to five years we found that children in the FNI group had significantly higher levels of RSA compared to the SC group (Welch et al., 2020a). In addition, RSA increased more rapidly in FNI children between infancy and the 4 to 5-year follow-up time point. Remarkably, we also found that mothers in the FNI group also had increased vagal tone at the 4–5 year follow-up. These results suggest that FNI-NICU leads to healthier long-term autonomic regulation in both mother and child.

These and previous findings strongly suggest that facilitating early nurturing interactions and emotional connection between preterm infants and their mothers is a practicable and effective means of optimizing postnatal development in preterm infants.

## Theoretical advances

Many new theoretical concepts and constructs came out of the FNI-NICU trials. All of these involve the autonomic nervous system, which controls function of internal organs, including the heart. The theories and constructs describe the special neurobiological relationship between birth mother and newborn infant. Attachment theory, first developed by Bowlby (1988) and later systematized for scientific investigation by Mary Ainsworth (Bretherton, 1992; Ainsworth et al., 1971), emphasizes the central nervous system of the infant. The attachment model posits that the baby is born with a set of behaviors toward a *mother-figure*, which are motivated by self-interest and survival factors and which do not necessarily need to be learned or reinforced by the birth mother (Ettenberger et al., 2021; Guy-Evans, n.d.). As theorized, the mother is not substantially differentiated from other caregivers. In contrast, the autonomic model emphasizes the autonomic nervous systems of the infant and mother and holds that each baby is born with a singular dyadic *autonomic emotional relationship* to the birth mother, whether the birth is term or preterm, or whether the birth is single or multiple. Following birth, mutual autonomic learning, adaptation and reinforcement between the mother and baby plays a key role in determining the developmental trajectory of the infant.

**Figure 5:**
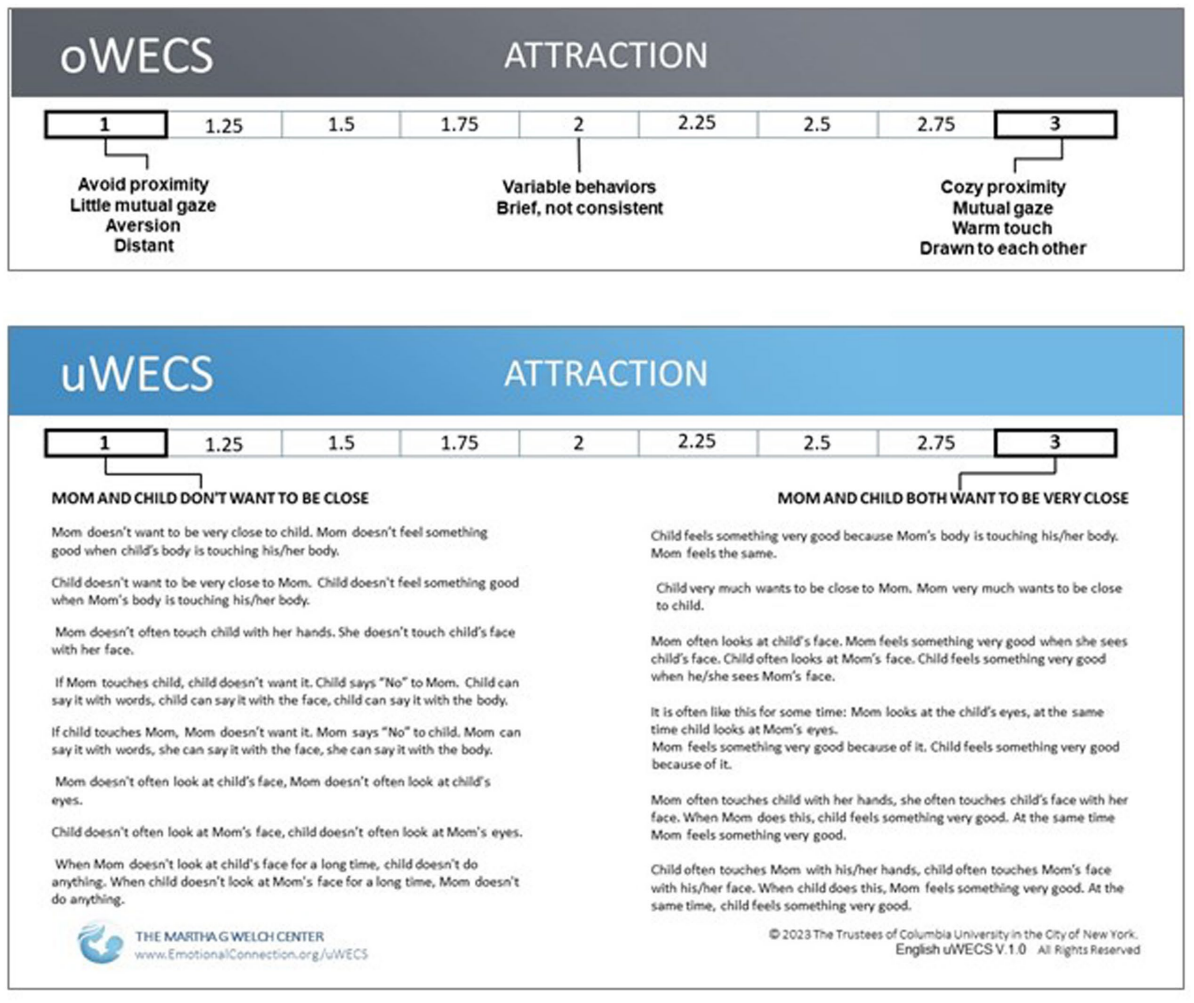
Comparison of the descriptors of ‘*Attraction*’ on the **(A)** Original Welch Emotional Connection Screen (oWECS) and **(B)** the Universal Welch Emotional Connection Screen (uWECS). The descriptors on the oWECS turned out to be confusing to coders and difficult to translate into other languages. This led to the development of the uWECS. Note that while longer than the original descriptors, the uWECS only employs approximately 65 ‘universal’ words common to all languages, making it easily accessible worldwide.

The basics of the autonomic theory were articulated in the FNI study protocol (Welch et al., 2012), but subsequently the theory has been more fully explicated (Welch and Ludwig, 2017a; Ludwig and Welch, 2019; Welch and Ludwig, 2017b; Welch, 2016).

## Biological mechanisms

### Autonomic socioemotional reflex

Measuring the mother and infant relationship in terms of autonomic emotional connection (AEC) required rethinking the biological mechanisms mediating the phenomenon. Conventional constructs, such as attachment and bonding, focus on conscious and unconscious cortical learning mechanisms. In contrast, AEC theory posits that mother/infant emotions are controlled by highly conserved primitive learning mechanisms operating outside of consciousness. AEC theory proposes that specialized primary autonomic (i.e., cardiac) reflexes form between mother and fetus during gestation via autonomic learning or conditioning (Ludwig and Welch, 2020). Due to their critical role in infant and child development, we have termed the mechanism the *autonomic socioemotional reflex (ASR)* (Ludwig and Welch, 2020). The ASR mechanism provides a biological explanation for mother-infant behaviors that are measured on the uWECS.

**Figure 6:**
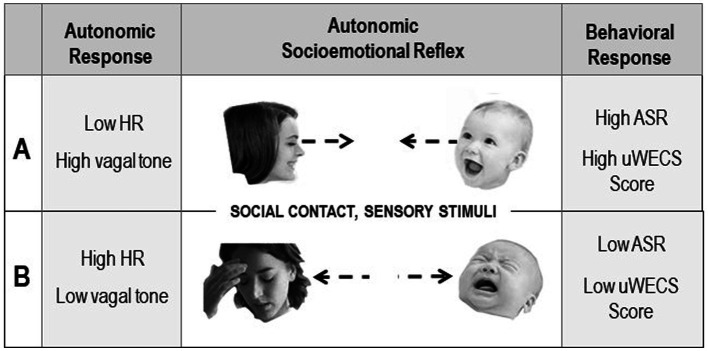
Diagram illustrating the Autonomic Socioemotional Reflex (ASR) and the hypothesized relationship between behaviors measured with the uWECS and autonomic physiology in two polar conditions. The central panels show opposite responses triggered by sensory stimuli resulting from close face to face social contact. Condition A shows a positive autonomic and behavioral response. Condition B shows a negative response. The left panels show the autonomic physiology in the two conditions, as measured by heart rate and vagal tone. The right panels show the Autonomic Socioemotional Reflex (ASR) behavioral response, as measured with the Universal Welch Emotional Connection Screen (uWECS).

These ASR reflexes are ‘conditional’ in the sense that the behaviors associated with the ASR depend on environmental (in this case ‘social’) conditions. The ASRs account for subconscious so-called ‘*instinctive’* or *‘innate’* mother-infant behaviors following birth. For instance, in a normal gestation and birth, gestational autonomic conditioning leads to adaptive social signaling between the mother and baby that assures ‘approach’ behavioral responses. In preterm birth, however, when the critical social signaling is disrupted between the two, the condition can result in adversely conditioned *‘avoidant*’ reflex behaviors (see Figure 6).

There is evidence that the ASR is common to other mammalian species. In fact, the idea was inspired by a phenomenon originally reported by Pavlov in 1925 when he described how the emotional relationship between a dog and trusted master profoundly impacted the dog’s autonomic function and behavior (Gantt et al., 1991). Pavlov termed this phenomenon a conditional ‘cardiac’ or ‘social’ reflex (Gantt, 1964). The phenomenon was later shown by Pavlov’s disciples to exist between many species, including humans (Gantt et al., 1991). We have extended this basic concept to the mother-infant relationship.

The ASR is a special case of the highly conserved *orienting reflex* (Ludwig and Welch, 2022). Dysfunctional orienting is highly correlated with socioemotional pathologies in infants and children, including social fear, anger, anxiety, depression and autism (Hedger et al., 2020). Orienting stems from activation of highly conserved autonomic defensive and appetitive motivational systems that evolved to sustain life (Boecker and Pauli, 2019), assuring the survival of species (Ludwig and Welch, 2022; Twilhaar et al., 2020). In this respect, the ASR orienting phenomenon in humans does not differ significantly from other species, from which it was conserved.

### Autonomic conditioning

Preterm birth is associated with cognitive learning difficulties, but the underlying mechanisms of these difficulties remain largely unclear (Twilhaar et al., 2020). The newborn infant’s environment, including support of the mother, is well known to be crucial to the infant’s survival. Yet how the environment influences behavior during this critical period remains unclear (Hunter et al., 2023). The FNI findings collectively suggest that focusing the interventional on subconscious ‘autonomic’ mother-infant co-conditioning offers a new way to optimize brain development and function, one that elevates the importance of relationship and co-regulation over many current efforts to develop self-regulation and independence.

Once the socioemotional signaling (i.e., social cueing) between the infant and mother is disrupted, as happens with premature birth, adverse autonomic conditioning of the dyad’s primitive ASRs ensues. As a result, dyads display social behaviors described as ‘avoidant’ on the uWECS (e.g., autonomically dysregulated and emotionally disconnected). This is a common situation in the NICU. It was the case with both groups at the beginning of the FNI-NICU trials. We theorized that FNI overcame the maladaptive relationship between mother and infant, by facilitating adaptive ‘*autonomic conditioning*’ of the ASRs during the mother-infant calming sessions. We theorize the mother’s autonomic emotional expression (AEE) during calming sessions acted as important ‘*signaling cues*’ and triggered the adaptive ASR orienting. In physiological terms, we can say that the maladaptive ASR produced by premature birth was ‘*counter-conditioned*’ according to Pavlov’s rules of autonomic conditioning (Gantt, 1964).

FNI-NICU focused on restoring the dysregulated autonomic states of mother and infant. Until recently, the consensus in neuroscience was that emotional behavior is controlled by the brain via top-down neuronal pathways and mainly conscious mechanisms (Krone et al., 2017). Over the past decade, however, evidence has emerged that suggests cognitive behavioral *states*, such as mood, motivation, attention, and arousal, are profoundly impacted by environmental signaling that involves integration of activity in the brainstem. Such behavioral states are modulated by the autonomic nervous system and the brainstem, which are activated in response to environmental challenges (Ludwig and Welch, 2022; Sara and Bouret, 2012).

Because of the fragility and medical condition of the preterm infant, it is common for the mother to feel helpless that her baby cannot respond to her. The opposite is true. By the third term of gestation, the autonomic neuronal wiring of the infant is fully in place to prompt cardiac orienting and attentional reflexes to the mother’s autonomic emotional expression.

Human hearing develops progressively during the last trimester of gestation. Near-term fetuses can discriminate acoustic features and process complex auditory streams on communication (Granier-Deferre et al., 2011). Newborns prefer their mother’s voice over other voices and perceive the emotional content of messages conveyed via intonation contours in maternal speech (“motherese”) (Mampe et al., 2009). A study showed that three weeks of prenatal exposure to a specific melodic contour affects infants ‘auditory processing’ or perception, i.e., impacts the autonomic nervous system at least six weeks later, when infants are one month old (Granier-Deferre et al., 2011). A systematic review of studies between 1966 and 2013 showed that motherese functions developmentally to communicate affect, regulate infants’ arousal and attention, and facilitate speech perception and language comprehension (Saint-Georges et al., 2013).

### Autonomic co-regulation

We make the case that FNI_NICU changed the mother-infant relationship and changed the infant’s and mother’s autonomic regulation. Indeed, our findings support this claim (Porges et al., 2019; Welch et al., 2020a; Ludwig et al., 2021). The idea of a connection between autonomic physiology and social relationship is not new. Darwin and Pavlov studied it (Ludwig and Welch, 2019). More recently it has become a focus of parent-infant and parent–child biobehavioral research (Palumbo et al., 2017). Various terms have been used to describe the social phenomenon: Physiological synchrony (Xu et al., 2024), biobehavioral synchrony, Concordance, Parent-infant synchrony (Feldman et al., 2011), Biological synchrony, Linkage, Physiological relatedness (van Puyvelde et al., 2015), Attunement, Covariation, Emotional engagement, Entrainment, Inter-individual synchronization, Physio-behavioral coupling, among others.

We refer to this phenomenon as ‘autonomic co-regulation’, by which we mean that the two autonomic nervous systems are inter-dependent, as opposed to independent. This interdependency starts *in utero* and continues after birth and is tied to survival and motivation and mental and physical health throughout development.

We theorize autonomic co-regulation with the mother to be the primary biological need of the baby. It is a function that carries with it individual and species survival value. When the mother is not available following birth, other family members can substitute, including the father. Family members can always play a critical role in the mother’s emotional and physical support, and in some cases the infant’s survival and development. Adoptive parents can also play a critical role in the development of the infant/child (Welch et al., 2006). However, if there is one key point we want all readers to take away from the FNI-NICU trials, it is that *the autonomic co-regulatory relationship between the mother and infant needs to be assessed, supported and treated in the NICU prior to discharge*.

Such autonomic co-regulation has been well-documented by the animal studies of FNI collaborator Myron A. Hofer. Hofer concluded that “in human development, early regulatory interactions may provide a bridge between biological and psychological processes in the development of our earliest mental representations” (Hofer, 1994). Indeed, our FNI findings lend support to Hofer’s hypothesis.

While we have proposed such a theory of change for FNI (Welch et al., 2020a), which includes experiencing mother infant autonomic co-regulation during calming sessions, establishment of mother/infant AEC, and counter conditioning adverse NICU experiences, we did not test this theory in the first RCT of FNI. Thus, explicating this underlying mechanism for early interventions remains a gap in the field. We are analyzing data from the FNI replication trial, which included repeated acquisition of mother and infant Heart Rate and RSA to better quantify mother/infant co-regulation over time as an index of autonomic conditioning. In another study we assessed emotional connection using the uWECS to test the hypothesis that emotional connection underlies effectiveness of the intervention.

## Behavioral constructs and assessment

### Autonomic emotional connection

The autonomic emotional connection (AEC) is a novel behavioral construct that describes the mutual behaviors of mother and baby that are triggered by the autonomic nervous system of each. There was no such construct prior to the FNI trials. Behaviors triggered by autonomic state require special assessment to differentiate them from psychologically triggered behaviors. In the first FNI trial, we had to use conventionally validated assessment tools that are based on conventional ‘attachment’ and ‘bonding’ theory and constructs (Pathak et al., 2023). Such tools measure separate *psychological* behaviors of infant and mother but not the dyad’s mutual contingent behaviors.

A recent review of the literature confirms that despite the emerging body of literature on mother-to-infant ‘bonding’ and the associated variables, there is a lack of consensus on the definition of the bonding construct, as well as a lack of a comprehensive conceptual framework of antecedents and consequences of bonding that would guide empirical work (Nakic Rados et al., 2023).

As our research progressed, we observed the same phenomenon Welch had seen in her prior clinical work: significant positive changes in the emotional relationship between the mother and the baby, which were not reflected in the conventional assessment tools. This led to an effort to validate a new instrument that would track the state of and then the change in the mother–child autonomic emotional relationship.

### Emotional connection and autonomic ‘state’

The behaviors measured by the uWECS are theorized to correlate with the dyad’s mutual autonomic ‘*states*’ at the time of observation, as opposed to their more or less fixed individual personality ‘*traits*’. This assumption produced new testable hypotheses regarding mother/infant approach-avoidance behaviors and the correlation between dyadic emotional behaviors (as measured on the uWECS) and autonomic physiology, such as heart rate and heart rate variability. For example, we could test the hypothesis that avoidant AEC behaviors (low uWECS score) correlate with higher HR and lower HRV. Conversely, AEC behavior (high WECS score) should hypothetically correlate with lower HR and higher HRV.

Being able to correlate behavioral data (e.g., uWECS scores) with autonomic state data has several benefits. First, it makes the rapid ‘in the moment’ uWECS assessment a true relational health biomarker. The uWECS assessment in the NICU provides a simple measure to identify those dyads at risk for behavioral and developmental problems and a means of tracking progress or lack thereof throughout the NICU stay and at follow-up visits.

Second, being able to make a rapid uWECS assessment in the NICU means the clinician can act on that information. Steps can be taken by in the NICU to help change a dysregulated to a co-regulated state. Again, the uWECS assessment offers a simple way to determine whether an intervention has successfully changed the dyad’s autonomic state.

### The universal Welch emotional connection screen (uWECS)

We needed to create a new assessment tool to measure AEC. Based on her clinical observations, Dr. Welch was convinced that a few key mother–child behaviors could form the basis for assessing the autonomic emotional relationship between mother and infant and could be used to identify dyads at risk for behavioral and developmental problems. Using data from the first FNI trial, we validated the original WECS (oWECS) against a standard social engagement coding software (Hane et al., 2024) (See Figure 5, Panel A).

The oWECS was validated in the FNI sample at the 4-month follow-up. In addition to demonstrating the validity and feasibility of the WECS quick screening tool with labor-intensive observational software, the WECS also demonstrated construct validity. Dyads measured high on AEC by the oWECS were characterized by higher mutual approach behaviors and more optimal autonomic regulation following the stress of the maternal still-face disruption (Hane et al., 2019).

The process validated a subset of four measurable mutual mother/infant behaviors: *attraction, vocal communication, verbal communication and sensitivity/reciprocity*. As predicted, the four behavioral subsets correlated with the preterm infant’s autonomic state physiology (cardiac response to stress) (Hane et al., 2019). These findings support the idea that during close face to face contact the mother/infant behaviors, as measured by the oWECS, are a mirror of the dyad’s autonomic physiology. This makes the oWECS a new way of assessing the dyad’s emotional relationship at the time of observation.

The oWECS was a key clinical and research advance validated with the FNI trials. The oWECS offers a fast, simple and actionable behavioral assessment tool that correlates with autonomic physiology, so the tool can be used to determine risk for impaired socioemotional and relational health throughout development. A validation study of full term infants showed that ratings on the oWECS at infant age six months are associated with child behavioral problems at age three years (Frosch et al., 2019). An oWECS study in a pediatric resident training course found the instrument improved residents’ attitudes, self-efficacy, and perceived professional norms pertaining to early relational health. The oWECS improved accuracy in recognizing the dyadic AEC (O'Banion et al., 2021).

While employing the oWECS in FNI studies globally, confusion arose over the English language words used to describe the behavioral subscales. Therefore, providing a universally accessible language to describe the positive and negative opposite behaviors associated with the subscales became an imperative. This was especially true because mother/infant AEC is theorized to be a foundational building block of all human relationships, regardless of language or culture. A chance encounter with a linguist, author Ulla Vanhatalo, working with author Cliff Goddard, an expert in the field of Natural Semantic Metalanguage (i.e., how meanings are packaged and expressed differently in different languages) led to a two-year collaboration, during which the English oWECS was translated into the ~65 words of universally clear, explicit translatable language (Goddard et al., 2018). The resulting version is referred to as the Universal WECS (uWECS). The uWECS has replaced the oWECS as the recommended assessment tool (See Figure 5, Panel B).

The uWECS has fulfilled the goal of an easily translatable screen. Thus far, it has been translated from the original English into Akan, Arabic, Chinese, Finnish, German, Turkish, Polish, Russian and Spanish with few difficulties. The advantage of the uWECS is that it requires no.

training beyond the form itself. In fact, a recent study showed that scores by coders reading only the uWECS descriptions were highly correlated with scores by coders trained on the oWECS. Further, the oWECS has high criterion-related validity in assessing the efficacy of nurture-based intervention (Hane et al., 2024). Results indicate the uWECS can produce relatively fast reliability among diverse coders. Easy translatability allows non-English speakers to assess mother-infant or mother/child autonomic emotional connection to improve pediatric relational health. It serves as both an assessment tool and a guide for parent-facing clinicians and parents.

## Theory of change

### Calming cycle theory

*Calming cycle theory* provides a novel explanation of how a maladaptive mother/infant emotional relationship can be changed to adaptive following birth (Welch, 2016). Conventional wisdom has held that instinctive or innate mother-infant behaviors are inherited and to some degree ‘*genetically*’ fixed at birth. A growing consensus holds that behavior can be influenced ‘*epigenetically*’ by the environment, prenatal as well as postnatal (Ludwig and Welch, 2019). Both positions assume, as Darwin believed, that emotional behavior is self-regulated or self-controlled via conscious higher brain function within the central nervous system. Accordingly, many NICU care strategies have aimed at getting the infant to self-regulate their physiology and emotional behavior, in line with attachment and bonding theory, which still drives many current therapies. There are notable exceptions that promote mother/infant ‘*co-regulation*’, such as kangaroo mother care (Arya et al., 2021; Adejuyigbe et al., 2023), or developmental care (White et al., 2023; Lehtonen et al., 2017). However, there is no consensus regarding the mechanisms involved.

A central tenet of calming cycle theory is that autonomic co-conditioning between mother and fetus during gestation and the postnatal period promotes co-regulation of autonomic states and emotional connection, which underlie improved socioemotional function over time. According to calming cycle theory, the autonomic states and behavior of mother and infant/child are co-regulated via a subcortical autonomic conditioning mechanism. After repeated calming, paired with sensory contact between mother and infant (i.e., a calming cycle), a conditioned autonomic calming reflex is triggered upon each further contact. This co-regulation phenomenon manifests itself behaviorally as mother–infant AEC and correlates with a high uWECS score. If practiced regularly, the calming cycle leads to less dysregulation and stronger emotional connection. In turn, the deeper the emotional connection provides a faster way to resolve autonomic dysregulation when it occurs.

The term “co-regulation” has been used with various definitions and connotations in animal and human research to describe co-dependency of physiological and behavioral activities between humans and even with other mammals. However, when the term has been used in reference to behavior, the process has invariably been discussed within a psychological framework (Butler and Randall, 2013). That is, cognitive mechanisms have conventionally been assumed to account for the effects of social interaction. Mother and infant systems have long been considered to be separate and independent (Guo et al., 2015). More recently, interest has grown in cognitive EEG ‘hyperscanning’ techniques, which are used to measure brain activity from more than one participant simultaneously, to understand the neural underpinnings of social interactions (Czeszumski et al., 2020).

Our interest centers around the assessment of autonomic co-regulation of the mother and infant’s autonomic nervous systems, which we posit are dynamically and continuously linked separately from the central nervous system. Accordingly, calming cycle theory holds that the dramatic impact of FNI on brain development reported above was mediated by co-conditioned changes in the dyadic autonomic nervous systems during and following the intervention.

## Is FNI-NICU scalable?

Scaling the exact FNI-NICU protocol is not practicable or scalable, nor was it our intention to do so. However, we believe the new tools and ideas from such as the trials are scalable. Implementing the mother-infant calming cycle, with its emphasis on autonomic emotional expression (AEE) requires little training and can be implemented within standard care or as part of other systems with little expense. The calming cycle can be implemented, for example, within the widely promoted KMC intervention or within Family Centered Care. The uWECS provides a simple monitoring tool for assessing the mother-infant autonomic emotional connection in as little as 3-min of observation. It is free and easily translatable into any language by a dual-language speaker.

The real barrier to scaling is resistance to new ideas. We note that the FNI study protocol was published in 2012, near the end of the first trial. This was not because there was uncertainty about method, goals and objectives at the beginning of the trial. The delay was mainly due to intense debates within our group over definitions, terminology and theory (Hofer, 2006). It took, for example, nearly a year of debate to settle on the word “nurture” to describe the intervention, which was at the time the object of derision among serious scientists. Another debate revolved around the hypothesis that autonomic conditioning was somehow involved in the intervention and that it could be tested. Despite ongoing debates over terminology and theory, by 2015 a consensus emerged in our group that FNI results require a rethinking of very long held assumptions about mother and infant behavior and the mother-infant relationship.

Part of validating the WECS instrument involved validating the theoretical association between specific behaviors between mother and infant and autonomic physiology. This involved obtaining simultaneous autonomic measures. Because of their association with early emotional health (Chiera et al., 2020), we chose heart rate (HR) and heart rate variability (HRV). We were able to show positive changes in infant HR (Ludwig et al., 2021) and HRV (Porges et al., 2019) following the intervention. Studies looking at the hypothesized pairing of changes in AEC as measured by the uWECS and maternal–infant/child physiological co-regulation are on-going.

## Summary

In retrospect, despite the optimism expressed when we started the FNI trials, it is understandable that many scientific experts were skeptical that anything useful would come out of them. The proposed rationale and theory supporting the FNI protocol was completely unfamiliar to reviewers. The trials challenged orthodox and conventional thinking at almost every step. In the end, it took very large and generous gifts over 25 years from a handful of private donors who believed in our research vision and cause to complete the research.

We have introduced many new constructs, theories, tools and biomarkers in this review, each of which will benefit from ongoing testing and validation. Hopefully, the evidence we assembled will lead to substantive changes in NICU care, including an emphasis on autonomic emotional connection and affording mothers and infants the time and support needed to establish and maintain their positive autonomic socioemotional reflex with each other. We are certain that the field will benefit from further exploring these promising evidence-based pathways, which could ultimately impact-real-world clinical practice (Hidalgo-Mazzei et al., 2018).

Finally, in closing we hope that the FNI trials inspire clinicians and researchers worldwide to consider acting on the lessons presented here. Imbedded in the material reviewed above is a scalable *blueprint for improving preterm birth outcomes*: (1) Encourage mother-baby autonomic emotional expression as a means of reconnecting the mother and baby. (2) Promote a daily calming cycle routine to strengthen and maintain an autonomic emotional connection. (3) Use the uWECS tool to monitor the autonomic emotional connection throughout development. (4) Use the uWECS as a clinical guide for families to improve autonomic emotional connection.
